# *Brucella abortus* Infection Promotes Mesenchymal Stem Cell Differentiation Toward Adipogenesis, Enhancing the Proinflammatory Profile

**DOI:** 10.3390/tropicalmed11050112

**Published:** 2026-04-23

**Authors:** Rosa Nicole Freiberger, Cynthia Alicia Marcela López, María Belén Palma, Cintia Cevallos, Franco Agustin Sviercz, Patricio Jarmoluk, Marcela Nilda García, Jorge Quarleri, M. Victoria Delpino

**Affiliations:** 1 Consejo Nacional de Investigaciones Científicas y Tecnológicas (CONICET), Laboratorio de Inmunopatología Viral, Instituto de Investigaciones Biomédicas en Retrovirus y Sida (INBIRS), Universidad de Buenos Aires (UBA), Buenos Aires 1121, Argentina; freibergernicole@gmail.com (R.N.F.); cevalloscintia@gmail.com (C.C.); quarleri@fmed.uba.ar (J.Q.); 2 Cátedra de Citología, Histología y Embriología, Facultad de Ciencias Médicas, Universidad Nacional de La Plata, La Plata 1900, Argentina; mbpalma@med.unlp.edu.ar (M.B.P.); mngarcia@med.unlp.edu.ar (M.N.G.); 3 Laboratorio de Investigación Aplicada a Neurociencias (LIAN), Fleni, Consejo de Investigaciones Científicas y Técnicas (CONICET), Escobar 1625, Argentina

**Keywords:** brucellosis, MSCs, osteoblasts, osteoarticular, adipocyte, leptin, lipid-droplets

## Abstract

The most common complication of active brucellosis in humans is osteoarticular injury. In the bone marrow microenvironment, mesenchymal stem cells (MSCs) can differentiate into either adipocytes or osteoblasts, and this balance is tightly regulated because an increase in adipogenesis may negatively affect bone formation and favor bone loss. The differentiation of MSCs into adipocytes or osteoblasts is tightly regulated by mechanisms that promote cell fate toward one lineage while repressing the other. Our study demonstrated that *Brucella abortus* infects MSCs but does not affect the deposition of organic and mineral matrix during osteoblast differentiation. However, the infection upregulates Receptor Activator of Nuclear Factor Kappa-B Ligand (RANKL) expression in osteoblasts, which may contribute to osteoclast activation and bone resorption. Conversely, *B. abortus* infection significantly influences adipocyte differentiation by modulating lipolysis, lipogenesis, and interactions between lipid droplets and mitochondria. This leads to increased cellular cholesterol levels and reduced intracellular triglycerides, accompanied by glycerol release. These changes result in more differentiated adipocytes and larger lipid droplets. Consequently, we observed increased IL-6 secretion and a higher leptin/adiponectin ratio. Importantly, these effects were independent of a functional type IV secretion system (T4SS), as purified *Brucella* DNA fully reproduced the adipogenic phenotype. Moreover, inhibition of TLR9—the primary sensor of bacterial DNA—significantly reduced the DNA-induced adipogenic response, demonstrating that adipocyte modulation is at least in part mediated through TLR9 signaling. In summary, *B. abortus* promotes MSC differentiation toward an inflammatory adipocyte phenotype. It involves a TLR-9-mediated DNA detection. It may contribute to osteoarticular injury and infection-associated bone resorption.

## 1. Introduction

Brucellosis is a widespread zoonotic infection worldwide, although its impact is markedly higher in developing countries [1]. The most common clinical complication of active human brucellosis is osteoarticular disease [2,3,4,5,6,7,8].

In addition to other sites, *Brucella* can persist in the human bone marrow [9,10]. In most patients, cellular alterations in the bone marrow improve or disappear after antibiotic treatment [9]. Moreover, transmission of brucellosis through bone marrow transplantation from apparently healthy donors has been reported [11]. These findings indicate that even when the bacterium is not detected in the bone marrow, it may persist, concealed within cells [12,13]. This observation suggests that the ability of *Brucella* to cause chronic infection may be linked to its persistence in the bone marrow.

Mesenchymal stem cells (MSCs) are among the key cellular components of the bone marrow microenvironment. They are multipotent precursor cells capable of differentiating into various stromal lineage cell types, including chondrocytes, adipocytes, and osteoblasts [14,15]. The differentiation of MSCs into adipocytes or osteoblasts is tightly regulated by mechanisms that promote commitment to one lineage while repressing the other [16]. For instance, osteoporosis, the most prevalent bone remodeling disorder globally, often exhibits heightened marrow fat content [17,18]. Indeed, increased bone marrow adiposity is commonly observed in many conditions associated with bone loss, such as aging and various pathological states [19,20,21].

The commitment of MSCs to either the osteoblast or adipocyte lineage depends on specific transcriptional regulators [22]. Among these, peroxisome proliferator-activated receptor gamma (PPAR-γ) and CCAAT/enhancer-binding protein (C/EBP) play critical roles in adipogenesis [23]. In contrast, Runt-related transcription factor 2 (RUNX2) acts as the master transcription factor for osteoblast differentiation [24]. This process is further regulated by PPAR-γ, which inhibits osteoblastogenesis by suppressing the transcription of RUNX2 [25].

Previous findings using murine cells indicate that *Brucella abortus* can modulate responses of adipocytes and osteoblasts, suggesting that both cell types could be involved in bone damage [26,27,28].

Therefore, we hypothesize that *B. abortus* interacts with MSCs and may promote adipocyte differentiation while reducing osteoblast differentiation; however, this process remains unclear and requires further investigation.

Here, we explore the interaction between *B. abortus* and MSCs during their differentiation into osteoblasts and adipocytes.

## 2. Materials and Methods

### 2.1. Bacterial Culture

*Brucella abortus* S2308, DsRed-expressing *B. abortus* S2308, or the isogenic *B. abortus virB*10 polar mutants (kindly provided by Diego Comerci, Universidad Nacional de San Martín (UNSAM), Buenos Aires, Argentina) were cultured for 18 h in 10 mL of tryptic soy broth supplemented with yeast extract (Merck, Darmstadt, Germany), under constant agitation (150 rpm) at 37 °C. Bacteria were harvested, and inocula were prepared as previously described [29].

To prepare heat-killed *Brucella abortus* (HKBA), the bacteria were washed five times with sterile phosphate-buffered saline (PBS), with each wash lasting 10 min. Subsequently, they were heat-inactivated at 70 °C for 20 min, then aliquoted and stored at −70 °C until use. The absence of bacterial growth on tryptose soy agar confirmed the complete loss of *B. abortus* viability. All manipulations involving live *B. abortus* were performed in biosafety level 3 facilities.

### 2.2. DNA from B. abortus

*Brucella abortus* DNA was isolated using the Wizard^®^ Genomic DNA Kit (Promega, Madison, WI, USA) in accordance with the manufacturer’s protocol. The concentration of the extracted DNA was determined using a spectrophotometer. Following the extraction process, a 100 µg aliquot of the DNA was treated with DNase I (1 U/mg DNA) (ZymoResearch, Beijing, China), as per the manufacturer’s guidelines. HKBA (2.5 × 10^9^ bacteria) was treated with 1 U of DNase in a final volume of 50 µL.

### 2.3. Isolation of Mesenchymal Stem Cells (MSCs)

The umbilical cords were preserved in α-Minimal Essential Medium (α-MEM, Gibco, Waltham, MA, USA) and handled following previously outlined procedures [30]. Essentially, each umbilical cord underwent a 5 mm fragmentation, with a sagittal incision exposing Wharton’s jelly. Removal of the umbilical blood vessels was meticulously conducted using clamps. Subsequently, the fragments underwent 2 or 3 washes with Dulbecco’s phosphate-buffered saline from Sigma-Aldrich (St. Louis, MO, USA) to eliminate residual blood. The section with the exposed jelly was then positioned face down at the bottom of a culture plate, and a minimum essential medium (α-MEM, Gibco) supplemented with 10% platelet lysate was added. The plates were incubated at 37 °C in a humid atmosphere containing 5% CO_2_, and medium was refreshed every 2 to 3 days 2 to 3 days. Expansion of umbilical cord-derived MSCs was typically observed between 10 and 14 days post-explantation, and cells were further amplified until reaching passage 2/3. MSCs were characterized by the expression of CD105, CD73, and CD90, and the absence of CD45, CD34, CD14, CD19, and HLA-DR molecules. For experimental purposes, MSCs were cultured in DMEM supplemented with 10% heat-inactivated fetal bovine serum (Gibco-BRL, Life Technologies, Grand Island, NY, USA), 100 U/mL of penicillin, and 100 mg/mL of streptomycin (complete medium) and were utilized until passage 5. This study obtained approval from the Comité de Bioética y Ética, Facultad de Ciencias Médicas, Universidad de Buenos Aires, Argentina (RESCD-2023-1291). Written informed consent was obtained from each mother before normal cesarean birth, and human umbilical cords were collected from discarded placentas.

### 2.4. Adipocyte Differentiation

Initially, MSCs were seeded at a density of 5 × 10^4^ cells per well in 24-well plates and allowed to reach confluence. Following this, the culture medium was replaced with adipocyte differentiation medium, comprising DMEM supplemented with 0.5 mM 3-isobutyl-1-methylxanthine (IBMX), 0.01 µM dexamethasone, 50 µM indomethacin, and 10 µg/mL human insulin, all sourced from Sigma Aldrich, St. Louis, MO, USA. Complete differentiation was achieved within 7 days.

### 2.5. Osteoblast Differentiation

Mesenchymal stem cells were seeded at a density of 5 × 10^4^ cells per well in 24-well plates and cultured until reaching confluence using a complete medium. Subsequently, complete differentiation of osteoblasts was achieved between day 14 and day 21 by culturing with a specific differentiation medium. This medium consisted of a complete medium supplemented with 10 mM β-glycerophosphate, 0.1 µM dexamethasone, and 50 µM ascorbic acid, all obtained from Sigma.

### 2.6. Brucella Infection of Cultured MSCs

Mesenchymal stem cells were seeded in 24-well plates and infected with *B. abortus* S2308, DsRed-expressing *B. abortus* S2308, or its isogenic *virB*10 mutant at a multiplicity of infection (MOI) equal to 100. After adding the bacterial suspension, the plates were incubated for 2 h at 37 °C in a 5% CO_2_ atmosphere. The cells were then thoroughly washed with DMEM to remove extracellular bacteria and incubated in a medium supplemented with 100 µg/mL gentamicin and 50 µg/mL streptomycin to eliminate any remaining extracellular bacteria. For the osteoblast and adipocyte differentiation experiments, the culture medium was replaced with osteoblast differentiation medium or adipocyte differentiation medium three days post-infection.

### 2.7. Stimulation with HKBA and DNA

Mesenchymal stem cells were stimulated with *B. abortus* DNA (0.1–1000 ng/mL), corresponding to DNA obtained from 1 × 10^4^ to 1 × 10^8^ bacteria, or with HKBA (1 × 10^6^ bacteria/mL). Three days after stimulation, the culture medium was replaced with adipocyte differentiation medium.

### 2.8. Preparation of Cellular mRNA and RT-qPCR

Real-time PCR was performed using SYBR Green as a DNA-binding fluorescent dye on a StepOne Real-Time PCR System (Applied Biosystems, Waltham, MA, USA). The primer sequences used for amplification are listed in Table 1.

All primer sets yielded a single product of the correct size. The amplification cycle for *PPARγ*, *CEBPα*, *CEBPβ*, *HSL*, *DGAT1*, *DGAT2*, and *FASN* was 95 °C for 15 s, 59 °C for 30 s, and 72 °C for 60 s. For *LPL*, *ATGL*, SREBP1, *SREBP2*, *Leptin*, and *AdipoQ* the cycle was 95 °C for 15 s, 60 °C for 30 s, and 72 °C for 60 s. Relative transcript levels were calculated using the 2^−ΔΔCt^ method using *β-actin* as a normalizer gene.

### 2.9. Measurement of Activator of Nuclear Factor Kappa-B Ligand (RANKL) and IL-6 Protein Expression

The expression of RANKL and IL-6 was assessed in culture supernatants using an ELISA kit (R&D Systems, Minneapolis, MN, USA, and BD Pharmingen, San Diego, CA, USA, respectively), following the manufacturer’s instructions.

### 2.10. Assessment of Calcium Deposition via Alizarin Red S Staining

After 14 and 21 days of osteoblast differentiation, cells were fixed with 4% paraformaldehyde (PFA) and stained with 2% (*w*/*v*) Alizarin Red S to assess calcium deposition, as previously performed in our laboratory [27]. The stained cells were then examined by light microscopy.

For quantitative analysis, the monolayers were detached using 10% (*v*/*v*) acetic acid heated to 85 °C for 10 min. The supernatants were then neutralized with 10% (*v*/*v*) ammonium hydroxide, and absorbance was measured at 405 nm using a microplate reader, following procedures previously established in our laboratory [27].

### 2.11. Assessment of Collagen Deposition by Sirius Red Staining

Collagen deposition was assessed at 14 and 21 days post-differentiation using Sirius red dye (Sigma-Aldrich, Buenos Aires, Argentina), as was previously described [27]. Before fixation, the cells were stained with the Sirius red dye reagent dissolved in saturated aqueous picric acid (0.1%) and observed under light microscopy. For quantitative analysis, the stained material was dissolved in 0.2 mL of 0.1 N sodium hydroxide, and the optical density (OD) was measured at 550 nm using a microplate reader.

### 2.12. Assessment of Adipocyte Differentiation Through Lipid Droplet Accumulation

To assess adipocyte differentiation, we used Bodipy 493/503 (Life Technologies, Carlsbad, CA, USA). Cells were cultured in 24-well plates, fixed with 10% formalin for one hour, and permeabilized with 0.3% Triton X100. Subsequently, lipid droplets were stained with 1 µg/mL of Bodipy 493/503 from Invitrogen. For nuclear visualization, DAPI (Thermo Scientific, Waltham, MA, USA) was applied as a counterstain. The prepared coverslips were mounted in a PBS-glycerin solution (9:1 *v*/*v*) and examined using a Zeiss LSM 800 confocal microscope (Jena, Germany). For each experimental set, ten microscopic fields per well were quantified, from three wells per condition.

### 2.13. Mitochondria Staining

To identify mitochondria, cells were incubated in the dark at 37 °C and 5% CO_2_ for 35 min with 100 nM MitoTracker™ Deep Red (MTDR, Invitrogen, CA, USA), which reflects live mitochondria presence [31]. Cells were fixed with 4% paraformaldehyde (PFA) in phosphate-buffered saline (PBS) for 10 min at room temperature. Nuclei were counterstained using DAPI (Thermo Scientific) for visualization. Confocal imaging was performed using a Zeiss LSM 800 confocal microscope (Zeiss, Jena, Germany). Mitochondrial mass was assessed by analyzing changes in the fluorescence intensity ratio.

### 2.14. Confocal Image Analysis

Images were acquired with a Zeiss LSM 800 confocal microscope, utilizing Zen System software version 3.6 for image capture. The FIJI software version 2.9.0 (ImageJ, National Institutes of Health, Bethesda, MD, USA) was employed to analyze and quantify both the number and size of lipid droplets (LD) present in the captured images. To assess the colocalization between LDs labeled with Bodipy 493/503 and mitochondria stained with the MitoTracker Deep Red probe, the Manders’ correlation coefficient was calculated from the confocal images using FIJI software [32].

### 2.15. Glycerol Determination

Glycerol release was determined in differentiated adipocytes according to Garland and Randle’s protocol with some modifications [33]. The cell culture medium was aspirated and replaced with phosphate-buffered saline (PBS) supplemented with 2% fatty-acid-free bovine serum albumin (BSA). The cells were incubated for 6 h at 37 °C. Following the incubation period, the supernatants were collected and analyzed using the TG Colour GPO/PAP AA enzymatic assay kit (Wiener, Buenos Aires, Argentina) in accordance with the manufacturer’s protocol. Absorbance readings were performed at 505 nm using a microplate reader.

### 2.16. Intracellular Concentration of Triglycerides and Cholesterol

The concentration of triglycerides was determined in a lysate of differentiated adipocytes treated with 1% Triton X-100 in PBS. The triglycerides were measured using a commercial enzymatic assay kit TG Colour GPO/PAP AA (Wiener, Buenos Aires, Argentina). The extraction buffer was used to extract the lipid droplets, and triglycerides were then converted to glycerol and fatty acids using the lipase enzyme. The OD value at 505 nm was used to measure the released glycerol. The intracellular cholesterol level was quantified by a cholesterol assay kit according to the manufacturer’s protocol using the enzymatic kit Colestat (Wiener, Buenos Aires, Argentina). The OD value at 505 nm was used to measure the presence of cholesterol.

The relative concentration was normalized with the concentration of total proteins, according to the Bradford method, using a Bio-Rad Protein assay kit.

### 2.17. TLR9 Inhibition

To determine whether the adipogenic effect induced by *Brucella* DNA was mediated through TLR9 signaling, MSCs were treated with the inhibitory oligodeoxynucleotide INH-ODN-18 during the adipocyte differentiation protocol.

Mesenchymal stem cells were pre-incubated with INH-ODN-18 (Sigma-Aldrich, St. Louis, MO, USA) at final concentrations of 0.1, 1, and 10 μM for 40 min at 37 °C before stimulation. After pre-incubation, cells were stimulated with purified *B. abortus* DNA (1000 ng/mL) or HKBA in a concentration comparable to a MOI of 100 (1 × 10^6^ bacteria/mL). The inhibitor was maintained throughout the differentiation period by adding INH-ODN-18 at each medium refresh.

The selected concentration range was used to assess the dose-dependent inhibitory effect on TLR9-mediated adipocyte differentiation. Vehicle controls (cells receiving medium ± vehicle without INH-ODN) and stimulus controls (stimulated in the absence of inhibitor) were included in all assays. All experimental conditions were processed in parallel and repeated independently.

The sequence of inhibitory oligonucleotides (INH-ODN)-18 used was: 5′-CCTGGATGGGAACTTACCGCTGCA-3′ (linear oligodeoxynucleotide with full phosphorothioate backbone).

### 2.18. Statistical Analysis

Statistical analysis was performed using one-way ANOVA. Multiple comparisons among all pairs of groups were performed using Tukey’s post hoc test, and comparisons against two groups were performed using Student’s *t*-test and Mann–Whitney test. Normality was assessed using the Shapiro–Wilk test. Each experiment was replicated in triplicate using distinct culture preparations across 3–7 independent occasions. Graphical and statistical analyses were performed using GraphPad Prism 8.0. Data were expressed as mean ± SD, with significance levels indicated as follows: * *p* < 0.01, ** *p* < 0.001, *** *p* < 0.0001 vs. NI.

## 3. Results

### 3.1. Intracellular Replication of B. abortus in MSCs Is Impaired by Osteogenic Differentiation and Reduced During Adipogenesis

**Figure 1:**
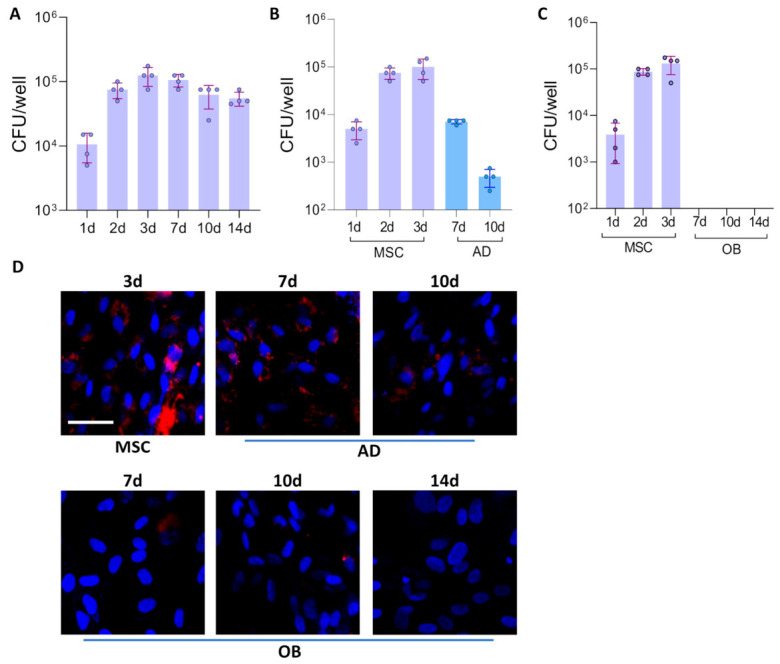
*Brucella abortus* replication in MSCs, adipocytes, and osteoblasts. Infection with *B. abortus* was performed at a multiplicity of infection (MOI) of 100, and CFU was determined at different times post-infection in mesenchymal stem cells (MSCs) (**A**), during adipocyte (AD) differentiation (**B**), and during osteoblast (OB) differentiation (**C**). Intracellular DsRed-expressing *B. abortus* in MSCs and during adipocyte and osteoblast differentiation was assessed by confocal microscopy. Nuclei were stained with DAPI (**D**). d (days post-infection). Data are expressed as mean ± SD obtained from 4 independent experiments.

Mesenchymal stem cells (MSCs) were infected with *B. abortus*. After 72 h of infection, some cells were cultured in osteoblast or adipocyte differentiation medium. The colony-forming units (CFU) of intracellular bacteria were determined at different times post-infection. As shown in Figure 1A, *B. abortus* infects and replicates in MSCs, reaching the highest level at 3 days post-infection, and these values did not differ significantly for at least 14 days post-infection. However, the levels of CFU declined after the initiation of adipocyte differentiation (Figure 1B). The osteoblast differentiation process completely abolished *B. abortus* to replicate in these cells (Figure 1C). These results were confirmed by confocal microscopy using *B. abortus* expressing DsRed (Figure 1D). These findings indicate that adipocyte and osteoblast differentiation modulate the intracellular survival of *B. abortus*.

### 3.2. Brucella abortus Was Unable to Modulate Matrix Deposition During Osteoblast Differentiation

**Figure 2:**
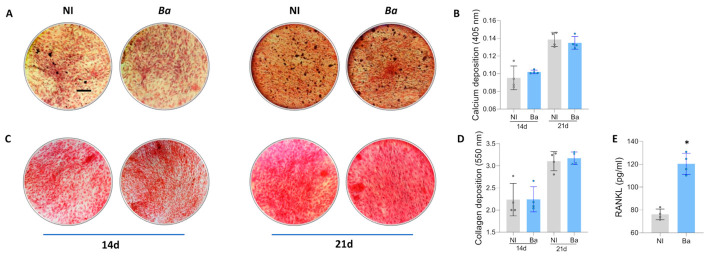
*Brucella abortus* was unable to modulate matrix deposition, but increased RANKL expression during osteoblast differentiation. Effect of *B. abortus* (*Ba*) infection on osteoblast differentiation. Representative microscopy images reveal calcium deposition by Alizarin Red S staining (**A**) and collagen deposition by Sirius Red staining (**C**) at 14 and 21 days post-differentiation. Spectrophotometric quantification of calcium (**B**) and collagen deposition (**D**). RANKL expression was determined in culture supernatants by ELISA at 21 days post-differentiation. NI (non-infected), d (days post-differentiation) (**E**). Ten microscopic fields per condition were quantified for each experiment. Scale bar: 100 µm. Data are expressed as mean ± SD measured in duplicate from 4 independent experiments. * *p* < 0.01 vs. NI.

Proper placement of the mineral matrix and the formation of the bone’s structural architecture depend critically on the accurate deposition of the organic matrix [34]. To investigate the impact of *B. abortus* infection on the deposition of organic and mineral matrices, MSCs were infected with *B. abortus*. Three days post-infection, the culture medium was switched to an osteoblast differentiation medium. The deposition of the mineral matrix, indicated by calcium deposits, and the organic matrix, indicated by collagen deposits, were evaluated at 14 and 21 days post-differentiation using Alizarin Red S (Figure 2A,B) and Sirius Red (Figure 2C,D) staining, respectively. The results revealed that calcium or collagen deposition among osteoblasts obtained from *Brucella*-infected MSCs remained unaltered at the observed time points.

### 3.3. Brucella abortus Induces Activator of Nuclear Factor Kappa-B Ligand (RANKL) Expression by Osteoblasts

Bone homeostasis is maintained through the balance between bone resorption by osteoclasts and bone formation by osteoblasts. RANKL is essential for the differentiation and activation of osteoclasts, playing a critical role in bone remodeling [35].

Our results indicated that osteoblasts derived from *B. abortus*-infected MSCs secrete higher levels of RANKL than osteoblasts derived from non-infected cells 21 days after differentiation (Figure 2E). Thus, *B. abortus* infection appears to disrupt bone homeostasis by promoting increased RANKL secretion, which could induce osteoclastogenesis.

### 3.4. Brucella abortus Infection Modulates Adipocyte Differentiation

**Figure 3:**
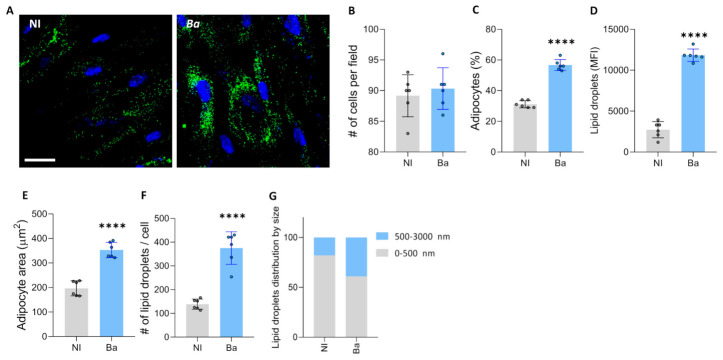
*Brucella abortus* modulates adipocyte differentiation. Effect of *B. abortus* (*Ba*) infection on adipocyte differentiation. Representative images of lipid droplets stained with Bodipy 493/503 at 7 days post differentiation (**A**). Quantification of the experiment performed in A (**B**–**G**). Number of cells per field (**B**). Percentage of adipocytes per field (**C**). Quantification of lipid droplets by mean fluorescence intensity (MFI) (**D**). Adipocyte area (**E**). Number of lipid droplets per cell (**F**). Lipid droplets size distribution (**G**). NI (non-infected). d (days post differentiation). Ten microscopic fields per condition were quantified for each experiment. Scale bar: 50 µm. Data are expressed as mean ± SD measured in duplicate from 6 independent experiments. **** *p* < 0.00001 vs. NI.

The differentiation of MSCs into osteoblasts or adipocytes is meticulously controlled by a combination of biological signals and chemical influences [20,21]. Therefore, to determine the effect of *B. abortus* infection on adipocyte differentiation, MSCs were infected with *B. abortus*, and after 3 days, the culture medium was replaced with adipocyte differentiation medium. A significant increase in adipocyte differentiation compared to uninfected cells was observed, as shown by the increases in adipocyte number, adipocyte size, and lipid droplets size evaluated at 7 days after differentiation (Figure 3).

**Figure 4:**
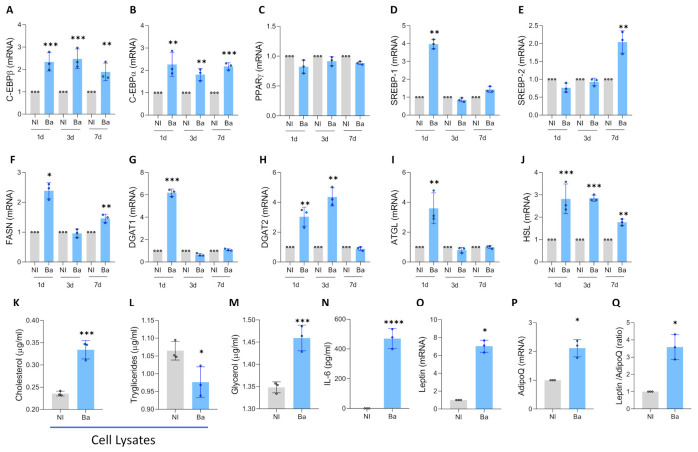
*Brucella abortus* modulates adipocyte mediators during its differentiation. Effect of *B. abortus* infection on *C/EBPβ* (**A**), *C/EBPα* (**B**), *PPARγ* (**C**), *SREBP1* (**D**), *SREBP2* (**E**), *FASN* (**F**), *DGAT1* (**G**), *DGAT2* (**H**), *ATGL* (**I**), and *HSL* (**J**) determined by RT-qPCR at day 1, 3, and 7 post differentiation. Cholesterol (**K**) and triglycerides (**L**) were measured in cell lysates. Glycerol release was measured in culture supernatants (**M**). IL-6 secretion was measured in culture supernatant by ELISA (**N**). *Leptin* (**O**) and *AdipoQ* (**P**) transcription were measured by RT-qPCR. *Leptin*/*AdipoQ* ratio (**Q**). Determinations from panels (**K**–**Q**) were performed at 7 days post-differentiation. Data are expressed as mean ± SD measured in triplicate from 3 independent experiments. * *p* < 0.01, ** *p* < 0.001, *** *p* < 0.0001, **** *p* < 0.00001 vs. NI.

*CCAAT/enhancer-binding protein (C/EBP)β* is expressed in the initial stages of differentiation and enhances the transcription of *C/EBPα* and Peroxisome proliferator-activated receptor gamma (*PPARγ)*. Mesenchymal stem cells were infected with *B. abortus* for 3 days and cultured in adipocyte differentiation medium. At 1, 3, and 7 days post-differentiation, the transcription of *C/EBPβ*, *C/EBPα*, and *PPARγ* was evaluated by RT-qPCR. An increase in *C/EBPβ* expression, along with concurrent transcription of *C/EBPα*, was observed (Figure 4A,B) without significant changes in *PPARγ* levels (Figure 4C).

### 3.5. Brucella abortus Infection Modulates Lipogenesis and Lipolysis During Adipocyte Differentiation

Adipocyte enlargement results from an increase in the size of lipid droplets. The enlarged lipid droplets demand more protein production to support the growth of plasma and lipid droplet membranes. Additionally, hypertrophic adipocytes encounter heightened demands for lipogenic, lipolytic, and endocrine activities, which increase cellular stress and necessitate the activation of growth signaling pathways [36].

*Sterol regulatory element-binding proteins (SREBP)-1* and *SREBP-2* are crucial transcription factors that regulate lipid metabolism during the differentiation of mesenchymal stem cells into adipocytes. *SREBP-1* is essential for activating genes involved in lipogenesis, including those coding for key enzymes in fatty acid and triglyceride synthesis. Its expression increases significantly during the first 24 to 48 h of differentiation. On the other hand, *SREBP-2* is involved in regulating cholesterol metabolism [37]. Our results indicate that *B. abortus* infection increases the transcription of *SREBP-1* on day 1 of differentiation and *SREBP-2* on day 7 post-differentiation (Figure 4D,E).

Accordingly, *B. abortus* infection induces an increase in lipogenesis, as revealed by the upregulation of *FASN* transcription at 1 and 3 days post-differentiation, and *DGAT1* and *DGAT2* at 1 and 3 days post-differentiation, respectively (Figure 4F–H). This is accompanied by lipolysis, evidenced by the increase in *ATGL* at day 1 post-differentiation and *HSL* at 1, 3, and 7 days post-differentiation (Figure 4I,J). As a result, at 7 days post-differentiation, *B. abortus* induces an increase in intracellular cholesterol compared to uninfected controls (Figure 4K). Additionally, a reduction in intracellular triglycerides was observed in cell lysates from cells infected with *B. abortus*, along with an increase in glycerol presence in culture supernatants (Figure 4L,M).

Adipocyte hypertrophy is associated with an inflammatory profile. Our results indicate that *B. abortus* infection induces an increase in IL-6 secretion (Figure 4N) and an increase in the leptin/adiponectin ratio (Figure 4O–Q). Together, these findings indicate that *B. abortus* modulates lipogenesis and lipolysis, leading to adipocyte hypertrophy with an inflammatory profile.

### 3.6. Brucella abortus Infection Increases Lipid Droplet-Mitochondria Interaction During Adipocyte Differentiation

**Figure 5:**
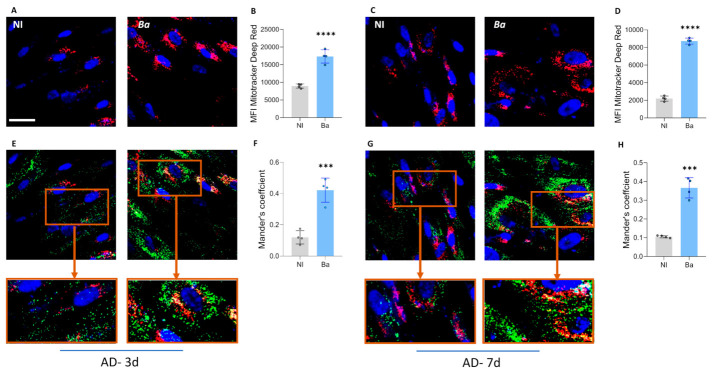
*Brucella abortus* infection increases lipid droplets and mitochondria interaction during adipocyte differentiation. Representative images showing mitochondria staining at 3 (**A**) and 7 (**C**) days post-differentiation. Quantification of median fluorescence intensity (MFI) of mitochondria amount using Image–J from images in A and C, at 3 (**B**) and 7 (**D**) days post differentiation. Lipid droplets stained with Bodipy 493/503 and mitochondria stained with MitoTracker Deep Red at 3 (**E**) and 7 (**G**) days post differentiation. Lipid droplets-mitochondria colocalization was quantified from 10 images using Mander’s overlap analysis at 3 (**F**) and 7 (**H**) days post-differentiation. Data are given as the mean ± SD measured in duplicate from 4 independent experiments. *** *p* < 0.0001, **** *p* < 0.00001 vs. NI.

Mitochondria bind to lipid droplets and regulate lipid storage and utilization [38]. Therefore, experiments were conducted to determine whether *B. abortus* infection increases interaction between mitochondria and lipid droplets. To this end, lipid droplets were stained with Bodipy 493/503, and mitochondria were labeled with MitoTracker Deep Red at 3 and 7 days after adipocyte differentiation. Our results indicate that *B. abortus* infection increases mitochondrial mass measured at 3 and 7 days post-differentiation (Figure 5 A–D). As expected and consistent with the increase in lipid droplet size in *B. abortus*-infected cells, a significant colocalization between lipid droplets and mitochondria was measured using Manders’ overlap analysis at 3 and 7 days post differentiation (Figure 5E–H). Our results indicate that *B. abortus* infection increases mitochondrial mass and the interaction between mitochondria and lipid droplets.

### 3.7. Brucella abortus Induces an Increase in Lipid Droplet Size Through a VirB- Independent Mechanism

**Figure 6:**
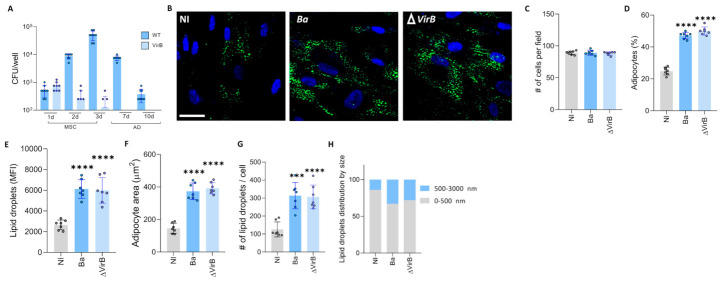
*Brucella abortus* increased lipid droplet size was independent of *Vir*B. Infection with *B. abortus* (*Ba*) and *B. abortus VirB*10 mutant (Δ*Vir*B) was performed at a multiplicity of infection (MOI) of 100, and colony-forming units (CFU) were determined at different times of mesenchymal stem cells (MSCs) and adipocyte (AD) differentiation (**A**). Effect of B. abortus and Δ*Vir*B infection on adipocyte differentiation (**B**–**H**). Representative images of lipid droplets stained with Bodipy 493/503 at 7 days post differentiation (**B**). Quantification of the experiment performed in A (**B**–**G**). Number of cells per field (**C**). Percentage of adipocytes per field (**D**). Quantification of lipid droplets by mean fluorescence intensity (MFI) (**E**). Adipocyte area (**F**). Number of lipid droplets per cell (**G**). Lipid droplet size distribution (**H**). NI (non-infected). d (days post differentiation). Ten microscopic fields per condition were quantified for each experiment. Scale bar: 25 µm. Data are expressed as mean ± SD measured in duplicate from 7 independent experiments. *** *p* < 0.0001, **** *p* < 0.00001 vs. NI.

The type IV secretion system (T4SS) *Vir*B is involved in the ability of different *Brucella* species to establish an intracellular replication niche and in the induction of inflammatory responses during *B. abortus* infection [39,40,41,42]. To this end, MSCs were infected with *B. abortus* and an isogenic *Vir*B mutant. Three days post-infection, the medium was replaced with adipocyte differentiation medium. The presence of intracellular bacteria was evaluated at 1, 2, 3, 7, and 10 days post-infection. These data indicate that the ability of *B. abortus* to replicate in these cells depends on the presence of a functional T4SS (Figure 6A). However, the modulation of adipocyte differentiation may not require a functional T4SS, as evidenced by the similar increase in adipocyte number, as well as in lipid droplets number and size, induced by both wild-type *B. abortus* and the *Vir*B mutant 7 days after differentiation (Figure 6B–H). These results indicate that *B. abortus* infection increases adipocyte number and size in a *Vir*B-independent manner.

### 3.8. Brucella DNA Recapitulates the Adipogenic Modulation Induced by B. abortus Infection

**Figure 7:**
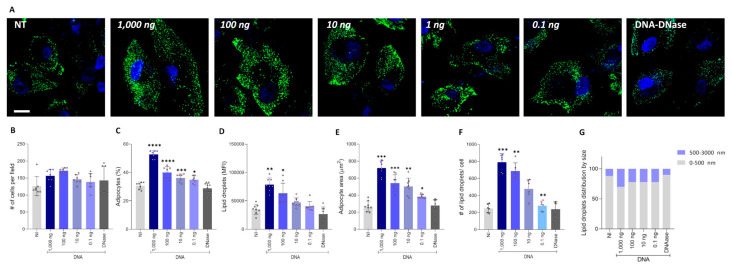
*Brucella abortus* DNA mimics the effects of *B. abortus* infection on adipocyte differentiation. Effect of stimulation with *B. abortus* DNA (1000 to 0.1 ng/mL) or 1000 ng/mL of DNA treated with DNase on adipocyte differentiation. Representative images of lipid droplets stained with Bodipy 493/503 at 7 days post differentiation (**A**). Quantification of the experiment performed in A (**B**–**G**). Number of cells per field (**B**). Percentage of adipocytes per field (**C**). Quantification of lipid droplets by mean fluorescence intensity (MFI) (**D**). Adipocyte area (**E**). Number of lipid droplets per cell (**F**). Lipid droplet size distribution (**G**). NT (non-treated). Ten microscopic fields per condition were quantified for each experiment. Scale bar: 25 µm. Data are expressed as mean ± SD measured in duplicate from 7 independent experiments. * *p* < 0.01, ** *p* < 0.001, *** *p* < 0.0001, **** *p* < 0.00001 vs. NI.

Bacterial DNA has been linked to adipocyte hypertrophy and an inflammatory profile [43,44]. As shown in Figure 7, *B. abortus* DNA influences adipocyte differentiation similarly to live bacteria, leading to an increase in adipocyte number, size, and lipid droplet accumulation observed seven days after differentiation. This effect was proportional to the amount of DNA present in the culture (Figure 7). Notably, when the highest dose of *B. abortus* DNA (1000 ng/mL) was treated with DNase I, the effect was abolished, confirming that bacterial DNA plays a direct role in modulating adipocyte differentiation. These results indicated that *B. abortus*-DNA modulates adipocyte differentiation in a dose-dependent manner.

### 3.9. Heat-Killed B. abortus (HKBA) Modulate Adipocyte Differentiation Through DNA

**Figure 8:**
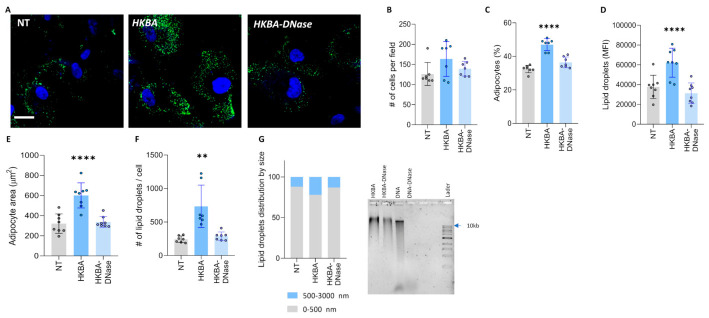
HKBA modulates adipocyte differentiation via DNA. Stimulation with heat-killed *B. abortus* (HKBA) at a concentration of 100 bacteria per cell. Representative images of lipid droplets stained with Bodipy 493/503 at 7 days post differentiation (**A**). Quantification of the experiment performed in A (**B**–**G**). Number of cells per field (**B**). Percentage of adipocytes per field (**C**). Quantification of lipid droplets by mean fluorescence intensity (MFI) (**D**). Adipocyte area (**E**). Number of lipid droplets per cell (**F**). Lipid droplet size distribution (**G**). NT (non-treated). Ten microscopic fields per condition were quantified for each experiment. Scale bar: 25 µm. Data are expressed as mean ± SD measured in duplicate from 7 independent experiments. ** *p* < 0.001, **** *p* < 0.00001 vs. NI.

To determine whether viable bacteria are necessary for modulating adipocyte differentiation from mesenchymal stem cells, we tested the ability of HKBA to mimic the effects of live bacteria. Similar to live *B. abortus*, HKBA induced an increase in adipocyte differentiation and lipid droplet size (Figure 8). The fact that HKBA also influenced the adipocyte differentiation process suggests that this phenomenon is mediated by a structural component of *B. abortus*.

To further investigate the role of *Brucella* genomic DNA in adipocyte differentiation, we treated HKBA with DNase I before using it to stimulate adipocyte precursors. Our results showed that DNase I-treated HKBA was unable to modulate adipocyte differentiation, as indicated by the comparable adipocyte number, size, and lipid droplet accumulation observed between treated and untreated cells. Our findings demonstrate that *Brucella* DNA plays a crucial role in modulating adipocyte differentiation, highlighting its potential impact on host metabolism.

### 3.10. Brucella DNA Modulates Adipogenesis Through TLR9 Signaling

It has been reported that TLR9 -the receptor responsible for recognizing bacterial DNA—is one of the TLRs most critically involved in *Brucella* infection [45]. To determine whether the adipogenic effect induced by bacterial DNA in our model depended on this pathway, MSCs were differentiated into adipocytes in the presence of the TLR9 antagonist INH-ODN-18.

**Figure 9:**
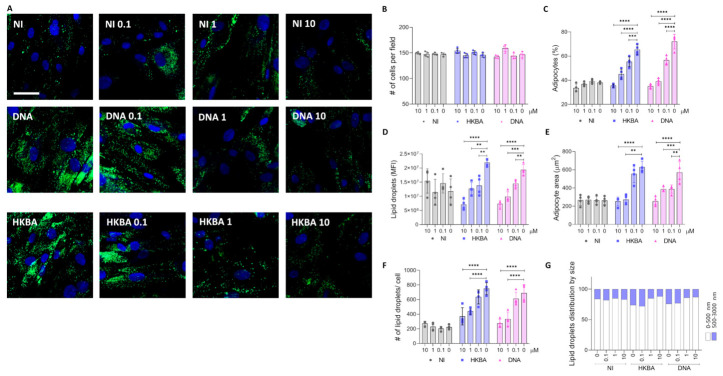
*Brucella* DNA modulates adipogenesis through TLR9 signaling. Stimulation with heat-killed *B. abortus* (HKBA), at a concentration of 100 bacteria per cell or *B. abortus* DNA (1000 ng/mL), was performed after pretreatment for 1 h with the TLR9 antagonist INH-ODN-18 at concentrations of 10, 1, and 0.1 µM. To determine if INH-ODN-18 per se affects adipocyte differentiation, non-treated (NT) cells were pretreated with INH-ODN-18. Representative images of lipid droplets stained with Bodipy 493/503 at 7 days post differentiation (**A**). Quantification of the experiment performed in A (**B**–**G**). Number of cells per field (**B**). Percentage of adipocytes per field (**C**). Quantification of lipid droplets by mean fluorescence intensity (MFI) (**D**). Adipocyte area (**E**). Number of lipid droplets per cell (**F**). Lipid droplet size distribution (**G**). NT (non-treated). Ten microscopic fields per condition were quantified for each experiment. Scale bar: 25 µm. Data are expressed as mean ± SD measured in duplicate from 7 independent experiments. ** *p* < 0.001, *** *p* < 0.0001, **** *p* < 0.00001 vs. NI.

As shown above, stimulation with *B. abortus* DNA markedly increased adipocyte number, cell size, and lipid droplet accumulation. However, preincubation with INH-ODN-18 significantly reduced these effects in a dose-dependent manner. The highest inhibitor concentration (10 μM) nearly abolished the adipogenic response to bacterial DNA, whereas intermediate (1 μM) and low (0.1 μM) concentrations produced partial inhibition (Figure 9A–F).

Importantly, INH-ODN-18 alone did not affect basal adipocyte differentiation, suggesting that the effects observed were related to TLR9 blockade. Similar results were obtained when cells were stimulated with HKBA, supporting the involvement of bacterial DNA in the adipogenic response. Taken together, these findings indicate that *B. abortus* DNA may promote adipocyte differentiation through a TLR9-dependent pathway, although additional bacterial components may also contribute.

## 4. Discussion

Osteoarticular disease is the most common clinical complication of active human brucellosis [2,3,4,5,6,7,8]. Bone marrow, located within the bone cavities, is a specialized tissue that plays essential roles in hematopoiesis, regulation of skeletal remodeling, and immune modulation [46]. Because *Brucella* can be detected in bone marrow during active infection [9,11], it is plausible that, in osteoarticular brucellosis, the bacterium may affect mesenchymal stem cell (MSC) differentiation and modulate the production of soluble mediators, thereby contributing to bone loss.

In the present study, we found that *B. abortus* infection of MSCs did not affect the deposition of organic or mineral matrix during osteoblast differentiation. This result differs from our previous observations, in which *B. abortus* inhibited osteoblast differentiation in murine preosteoblasts derived from calvaria and in the MC3T3-E1 cell line [27]. One possible explanation is that umbilical cord-derived MSCs have a high osteogenic potential [47]. In addition, MSCs are increasingly recognized as heterogeneous populations composed of distinct subpopulations with different cell surface marker profiles and functional properties [48]. Thus, one possibility is that *B. abortus* does not preferentially infect the MSC subpopulation most committed to osteogenic differentiation. Alternatively, the bacterium may infect and replicate before these cells enter the osteogenic program.

Although the deposition of mineral and protein matrix during osteoblast differentiation was preserved, *B. abortus* may still contribute to bone damage through another mechanism, namely by promoting osteoclastogenesis.

Activator of Nuclear Factor Kappa-B Ligand (RANKL), a homotrimeric molecule expressed on osteoblasts, is a central regulator of osteoclast differentiation and bone resorption [49]. Similar to other infections that increase RANKL expression [50,51,52,53], *B. abortus* infection of MSCs resulted in osteoblasts expressing higher RANKL levels than those differentiated in the absence of bacteria. Therefore, even in the absence of a direct effect on matrix deposition by differentiated osteoblasts, *B. abortus* may favor bone destruction by shifting the balance toward osteoclast activation.

A second major finding of this study is that *B. abortus* modulates adipocyte differentiation, increasing both adipocyte number and cell size. *CCAAT/enhancer-binding protein* (*C/EBP)β* is a key regulator of adipocyte development and lipid accumulation [54,55]. In agreement with this role, we observed increased *C/EBPβ* expression, together with concomitant induction of *C/EBPα*, in adipocytes differentiated from *B. abortus*-infected MSCs compared with uninfected controls. Likewise, adipocytes derived from infected MSCs showed an early increase in *SREBP-1*, a transcription factor that regulates lipid synthesis and uptake [56,57,58], along with increased expression of *fatty acid synthase* (*FASN*), *Diglyceride acyltransferase (DGAT)1*, and *DGAT2*. These findings indicate that *B. abortus* enhances the transcriptional program associated with adipogenesis and lipid accumulation.

Interestingly, *B. abortus* infection also increased lipolysis. Hypertrophic adipocytes are associated with increased lipolytic activity and reduced insulin responsiveness, which in turn lead to decreased glucose uptake, elevated basal lipolysis, and impaired stimulated lipolysis. As adipocytes enlarge through lipid droplet expansion, the demand for membrane and protein synthesis increases to sustain cell growth. Consequently, coordinated lipogenic and lipolytic activities are required to support these processes [59]. In this context, the increase in lipolysis observed in infected cells may contribute to the metabolic remodeling associated with adipocyte hypertrophy.

Mitochondrial mass is closely linked to the rate of lipid droplet expansion and may contribute to de novo lipogenesis and lipid droplet formation during early adipocyte differentiation [38]. Consistent with this, after *Brucella*-induced lipid droplet enlargement, we observed increased mitochondrial mass and a closer association between lipid droplets and mitochondria in adipocytes differentiated in the presence of *B. abortus* compared with uninfected controls. In parallel, glycerol derived from lipolysis can be recycled through phosphorylation by glycerol kinase and used as a substrate for triacylglycerol synthesis, suggesting an important role for glycerol recycling in adipocyte expansion [60]. In line with this mechanism, *B. abortus* induced glycerol release without significantly altering intracellular triglyceride levels at the time analyzed.

*Sterol regulatory element-binding protein (SREBP)-2* is primarily involved in cholesterol metabolism [57]. In enlarged adipocytes, this transcription factor can be activated in response to membrane cholesterol depletion to regulate genes involved in cholesterol biosynthesis [61]. Hypertrophic adipocytes are also characterized by a proinflammatory phenotype, including increased IL-6 production and a higher leptin/adiponectin ratio [62,63]. Therefore, the adipogenic program induced by *B. abortus* may not only promote cellular hypertrophy but also contribute to a local inflammatory environment that could further affect bone homeostasis.

Regarding the bacterial components involved in this response, the type IV secretion system (T4SS) encoded by the *vir*B genes is essential for intracellular replication of *Brucella* [39] and has also been implicated in the inflammatory response during *B. abortus* infection [41,64,65]. However, in our model, the T4SS was not involved in adipocyte differentiation. Instead, the effect of *Brucella* infection on adipogenesis was reproduced by HKBA, indicating that bacterial DNA is sufficient to trigger this response. This is consistent with previous studies showing that bacterial DNA contributes to adipose tissue inflammation and hypertrophy [43,44]. TLR9 has been identified as the most important TLR involved in *Brucella* infection, likely through recognition of bacterial DNA [45]. Although *Brucella* lipopolysaccharide (LPS) and lipoproteins also contribute to the immune response [66], their role in adipocyte differentiation was not determined until now. Nonetheless, we cannot exclude synergistic or context-dependent effects of these structural components. Further studies will be needed to determine whether TLR2-, TLR4-, or other DNA-independent pathways also participate in adipogenesis alongside TLR9. In agreement with this interpretation, inhibition of TLR9 with INH-ODN-18 markedly reduced the increase in adipocyte number, hypertrophy, and lipid droplet accumulation induced by HKBA and *B. abortus* DNA, supporting a key role for this receptor in the observed phenotype.

Overall, our results indicate that *B. abortus* infection does not impair osteoblast differentiation or function under the conditions tested. In contrast, it promotes adipogenesis and generates cells with a proinflammatory profile that may influence both osteoblast and osteoclast activity, thereby contributing to bone damage. In vitro models have provided valuable insights into the intracellular mechanisms involved [67], and the present study adds new information on *Brucella*-host interactions and advances our understanding of the cellular basis of bone involvement in brucellosis.

This study has some limitations. We used umbilical cord mesenchymal stem cells (UC-MSCs) instead of bone marrow mesenchymal stem cells (BM-MSCs) because UC-MSCs are more readily available and can be obtained non-invasively from tissue usually discarded after birth. In addition, we evaluated only *B. abortus* DNA as a bacterial stimulus involved in adipocyte hypertrophy, so the contribution of other *Brucella* components cannot be excluded. Further studies are needed to determine whether additional bacterial factors and Toll-like receptor pathways participate in this response.

## Figures and Tables

**Table 1:** Primer sequences.

Gene ID	Length (bp)	Sense Primer	Antisense Primer	Product Size (bp)
*Beta-actin (β-actin)*	1812	5′-CCTGGCACCCAGCACAAT-3′	5′-CGGGATCCACACGGAGTACT-3′	70
*Peroxisome proliferator-activated receptor gamma (PPARγ)*	1856	5′-GGCCGCAGATTTGAAAGAAG-3′	5′-GTTTGAGAAAATGGCCTTGTTGT-3′	64
*CCAAT/enhancer-binding protein (C-EBP)* *α*	2601	5′-CCAAGAAGTCGGTGGACAAGA-3′	5′-ATTGTCACTGGTCAGCTCCA-3′	143
*C-EBPβ*	2113	5′-TACTACGAGGCGGACTGCTT-3′	5′-CTGGTAGCCGAGGTAAGCG-3′	555
*Hormone-sensitive lipase (HSL)*	2791	5′-CATCTCCATTGGGCTGGTGT-3′	5′-ATCTCAAAGGCTTCGGGTGG-3′	279
*Adipose triglyceride lipase (ATGL)*	2416	5′-CAAGCGGAGGATTACTCGCA-3′	5′-CAAGCGGATGGTGAAGGACA-3′	210
*Fatty acid synthase (FASN)*	8459	5′-GCGTGGCCGGCTACTCCTAC-3′	5′-GTGTAGGCCAGTACGTAGGT-3′	136
*Diglyceride acyltransferase (DGAT)1*	3842	5′-CCGGACAATCTGACCTACCG-3′	5′-GGGATGTTCCAGTTCTGCCA-3′	410
*DGAT2*	3848	5′-GCCTGTGTTGAGGGAGTACC-3′	5′-CAGGGCCAGTTTCACAAAGC-3′	199
*Sterol regulatory element-binding proteins (SREBP)1*	4520	5′-GGGACCACTGTCACTTCCAG-3′	5′-TTCAAAGCTTCGACGCAGG-3′	105
*SREBP2*	4862	5′-ATGGGCAGCAGAGTTCCTTC-3′	5′-CGACAGTAGCAGGTCACAGG-3′	137
*Leptin*	3427	5′-GCTGTGCCCATCCAAAAAGTCC-3′	5′-CCCAGGAATGAAGTCCAAACCG-3′	135
*Adiponectin (AdipoQ)*	4593	5′-CAGGCCGTGATGGCAGAGATG-3′	5′-GGTTTCACCGATGTCTCCCTTAG-3′	92

## Data Availability

The raw data supporting the conclusions of this article will be made available by the authors, without undue reservation.

## Abbreviations

The following abbreviations are used in this manuscript:

MSCs	mesenchymal stem cells
α-MEM	α-Minimal Essential Medium
DMEM	Dulbecco’s Modified Eagle Medium
PBS	phosphate-buffered saline
IBMX	3-isobutyl-1-methylxanthine
RUNX2	runt-related transcription factor 2
IL-6	interleukin-6
RANKL	receptor activator of nuclear factor kappa B ligand
ALP	alkaline phosphatase
CFU	colony-forming units
C/EBP	CCAAT enhancer binding protein
HSL	hormone-sensitive lipase
ATGL	adipose triglyceride lipase
FASN	fatty acid synthase
DGAT	diglyceride acyltransferase
PPAR	peroxisome proliferator-activated receptor
